# Antiviral activity of nitazoxanide against *Morbillivirus* infections

**DOI:** 10.1016/j.jve.2023.100353

**Published:** 2023-11-02

**Authors:** Debora Stelitano, Simone La Frazia, Annalisa Ambrosino, Carla Zannella, Daniel Tay, Valentina Iovane, Serena Montagnaro, Anna De Filippis, Maria Gabriella Santoro, Matteo Porotto, Massimiliano Galdiero

**Affiliations:** a Department of Experimental Medicine, University of Campania “Luigi Vanvitelli”, via Santa Maria di Costantinopoli 16, 80138, Naples, Italy; b Department of Pediatrics, Columbia University Vagelos College of Physicians and Surgeons, 701 West 168th st, 10032, New York, NY, USA; c Center for Host–Pathogen Interaction, Columbia University Vagelos College of Physicians and Surgeons, 701 West 168th st, 10032, New York, NY, USA; d Department of Biology, University of Rome Tor Vergata, Via della Ricerca Scientifica 1, 00133, Rome, Italy; e Department of Agriculture Sciences, University of Naples “Federico II”, Via Università, 100-Portici, 80055, Naples, Italy; f Department of Veterinary Medicine and Animal Production, University of Naples “Federico II”, via Federico Delpino 1, 80137, Naples, Italy; g Institute of Translational Pharmacology, CNR, Via Fosso del Cavaliere 100, 00133, Rome, Italy; h Virology and Microbiology Unit, University Hospital “Luigi Vanvitelli”, via Santa Maria di Costantinopoli 16, 80138, Naples, Italy

**Keywords:** Nitazoxanide, Thiazolide, Measles, *Morbillivirus*, Canine distemper virus, Antiviral

## Abstract

The measles virus (MeV) and canine distemper virus (CDV) belong to the genus *Morbillivirus* of the *Paramyxoviridae* family. They are enveloped viruses harboring a non-segmented negative-sense RNA. Morbilliviruses are extremely contagious and transmitted through infectious aerosol droplets. Both MeV and CDV may cause respiratory infections and fatal encephalitis, although a high incidence of brain infections is unique to CDV. Despite the availability of a safe and effective vaccine against these viruses, in recent years we are witnessing a strong resurgence of *Morbillivirus* infection. Measles still kills more than 100,000 people each year, and CDV causes widespread outbreaks, especially among wild animals, including non-human primates.

No drugs are currently approved for MeV and CDV. Therefore, the identification of effective antiviral agents represents an unmet medical need. Here, we have investigated the potential antiviral properties of nitazoxanide (NTZ) against MeV and CDV. Antiviral activity was explored with live virus and cell-based assays. NTZ is a thiazolide that is approved by the FDA as an antiprotozoal agent for the treatment of *Giardia intestinalis* and *Cryptosporidium parvum*. Further, nitazoxanide and its metabolite tizoxanide have recently emerged as broad-spectrum antiviral agents. We found that NTZ blocks the MeV and CDV replication, acting at the post-entry level. Moreover, we showed that NTZ affects the function of the viral fusion protein (F), impairing viral spread. Our results indicate that NTZ should be further explored as a therapeutic option in measles and canine distemper virus treatment.

## 1 Introduction

The genus *Morbillivirus* belongs to the *Paramyxoviridae* family, a wide family of enveloped non-segmented negative-strand RNA viruses.^1^ *Morbilliviruses* are highly contagious, transmitted via respiratory droplets, and cause severe immunosuppression. The consequences of *Morbillivirus* infection in previously unexposed populations can be devastating, with high morbidity and mortality rates (e. g. historical measles outbreaks among Native Americans).^2^ The genus *Morbillivirus* includes important human and animal pathogens such as the measles virus (MeV), canine distemper virus (CDV), and peste des petits ruminants virus (PPRV). Known for the typical childhood rash it causes, measles infection can be extremely serious, with potential consequences including pneumonia and neurological complications. In addition, MeV has a very narrow host spectrum, affecting only humans. Measles infection starts in the respiratory tract with the infection of lymphocytes that express the SLAM receptor. Later in the course of infection, respiratory epithelial cells are infected through the interaction of MeV with the nectin-4 receptor expressed on the basolateral surface of these respiratory epithelial cells. Similarly, the CDV enters cells by exploiting the SLAM and nectin-4 receptors, infects lymphocytes, and causes a profound immunosuppression. While CDV is most commonly known to cause canine distemper in dogs, it has a broad host range that includes ferrets, foxes, coyotes, raccoons, lions, red pandas, and others.^3^ Distemper disease may occur with gastrointestinal, respiratory, or neurological symptoms.^4^ Moreover, the clinical picture depends on different factors, including the immune status, virus strain, and host age,^5^ and unvaccinated dogs and puppies are highly susceptible to CDV infection. Circulation of CDV in countries with high vaccination rates is due to the presence of wild animals such as raccoons, foxes, and ferrets, which act as natural reservoirs.^5^ CDV represents a global concern for several reasons, including its close relationship to the human MeV, its high cross-species transmission, and potential to spill from animals to humans.^6^ This last reason for concern—potential spillover of CDV from animals to humans—cannot be ignored despite notable differences in the amino acid sequences of human and canine SLAM receptors.^7^ In the recent past, CDV outbreaks have emerged among different mammal species, including macaques.^8^, ^9^, ^10^ Deadly epidemics have occurred in monkeys, indicating that this virus poses a threat to primates.^10^^,^^11^ Because SLAM and nectin-4 amino acid sequences are highly conserved between monkeys and humans,^11^^,^^12^ therefore a potential spillover of CDV to humans cannot be entirely excluded. Although an effective vaccine against MeV and CDV exists, most novel cases of infection have occurred among unvaccinated individuals, and so there is a public health imperative to develop an effective therapeutic strategy to complement vaccination.

Further, there are currently no antivirals licensed for the treatment of *Morbillivirus*, and while an efficacious MeV vaccine exists, MeV eradication is not in sight. Antiviral treatment for MeV could aid in the quest to eradicate the virus and to contain outbreaks.

Nitazoxanide (NTZ) is an FDA-approved thiazolide (licensed in the United States as Alinia®, Romark Laboratories) used in the clinic for treating gastroenteritis caused by *Cryptosporidium parvum* and *Giardia intestinalis* in children and adults.^13^

NTZ and its circulating metabolite tizoxanide (TIZ) have emerged as a new class of broad spectrum antiviral agents against different DNA and RNA viruses.^14^, ^15^, ^16^, ^17^ In particular, the thiazolides NTZ and TIZ were found to be effective against several RNA viral pathogens, including rotaviruses, coronaviruses, hepatitis C, influenza and parainfluenza viruses *in vitro*,^18^, ^19^, ^20^, ^21^, ^22^, ^23^ as well as in clinical studies.^24^, ^25^, ^26^

The proposed mechanisms of action include the alteration of the maturation of specific viral glycoproteins and the stimulation of the innate immune response. Interestingly, NTZ selectively blocks the maturation and intracellular transport of the hemagglutinin protein of human and avian influenza viruses,^20^^,^^21^ of the spike protein of seasonal and emerging human coronaviruses,^17^^,^^27^ as well as of the fusion protein (F) of the paramyxovirus Sendai virus (SeV) and respiratory syncytial virus (RSV).^22^

On the basis of these observations, we have investigated the antiviral activity of NTZ on *Morbillivirus* infection *in vitro.* In the present study, we show that NTZ inhibits both MeV and CDV replication; we also show that the activity of the viral fusion protein is reduced after nitazoxanide treatment, impairing viral spread. The results suggest that nitazoxanide may represent a valid therapeutic option in the treatment of *Morbillivirus* infections.

## 2 Results

### 2.1 Antiviral activity of nitazoxanide on measles and canine distemper virus infection

**Fig. 1:**
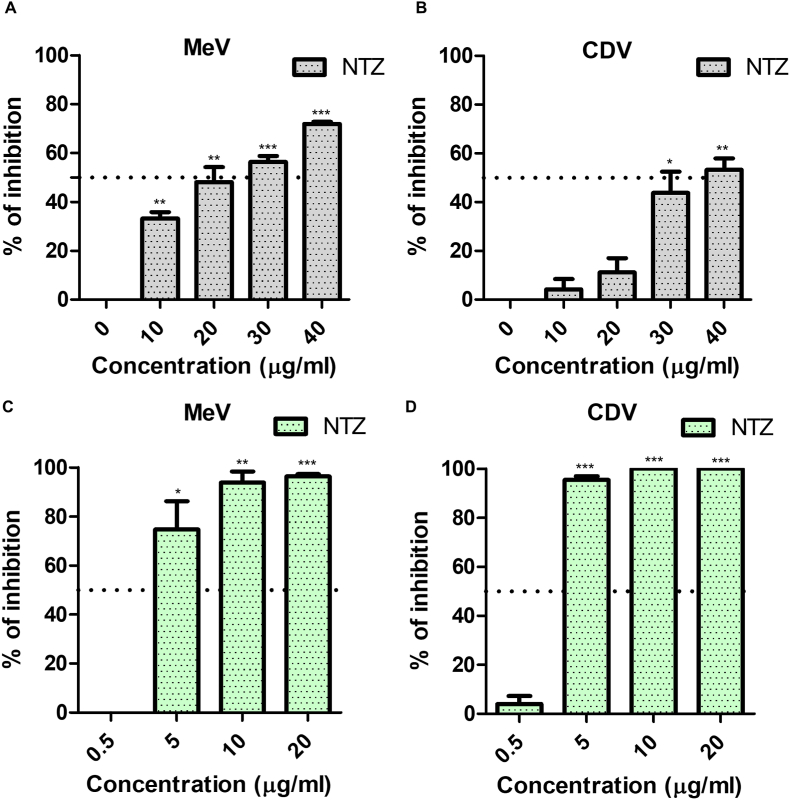
Antiviral activity of nitazoxanide (NTZ) against measles virus (MeV) and canine distemper virus (CDV). (A–B). MeV (A) and CDV (B) were incubated with the indicated concentrations of NTZ and added to Vero-hSLAM cells for 2 h to allow viral entry. After this time, infected cells were washed with PBS three times, and the medium containing carboxymethylcellulose was added. After 72 h, cells were fixed with 4% paraformaldehyde, and plaques were stained with crystal violet. Data represents the results from three independent experiments. (C–D) Vero-hSLAM cells were infected with 100 PFU/well of MeV (C) and CDV (D) viruses (96-well plate) and 2 h post-infection (p.i.), the medium was replaced with a complete medium containing different concentrations of NTZ. After 72 h, the supernatants were collected, and virus yield was determined by plaque assay. Data from three different experiments are shown. The error bars show the mean ± SEM.* p-value<0.05, **<0.01, ***<0.001. (For interpretation of the references to colour in this figure legend, the reader is referred to the Web version of this article.)

Despite the existence of a safe and effective vaccine, MeV and CDV continue to cause yearly outbreaks, and no approved antiviral therapy against *Morbillivirus* infection is currently available. We have therefore evaluated the antiviral potential of the thiazolide nitazoxanide against the *Morbillibiruses* MeV and CDV. Nitazoxanide was first tested in a 2-h co-treatment assay at concentrations ranging from 10 to 40 μg/ml (32–128 μM); under these conditions, antiviral activity was observed only at concentrations higher than 20 μg/ml (Fig. 1A and B).

**Suppl. Fig. 1:**
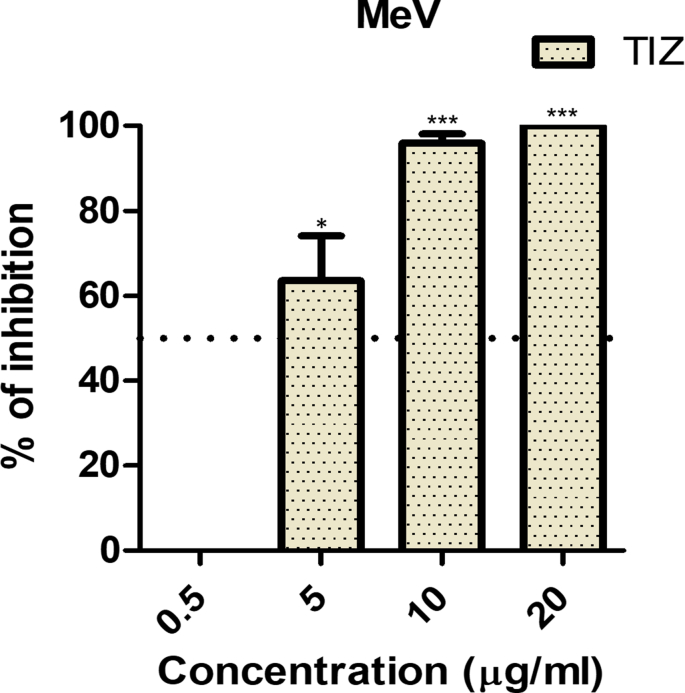
Antiviral activity of TIZ on MeV. Vero-hSLAM cells were infected with MeV 100 plaque forming unit (PFU)/well and 2 h p.i. the medium was replaced with a complete medium containing the different concentrations of TIZ. At 72 h p.i., the supernatants were collected and virus yield was determined by plaque assay. Data from three different experiments are shown. Error bars show the mean ± SEM. * p-value<0.05, ***<0.001.

Previously, it has been reported that NTZ blocks the replication of the paramyxovirus SeV, acting at a post-entry level.^22^ Hence, we speculated that the effect of NTZ during the infection step was most likely due to the 2 h effect of NTZ on the cells during the infection and that it would be more potent in post-infection assays. Vero-hSLAM cells were infected either with MeV or CDV and, after virus adsorption, the drug was added and kept for the duration of the experiment. Under these conditions, NTZ potently impaired MeV (Fig. 1C) and CDV (Fig. 1D) replication, with IC_50_s of 3.23 μg/ml and 2.83 μg/ml, respectively (Table 1). NTZ was not cytotoxic at the concentrations tested as confirmed by the MTT assay (Table 1). Moreover, tizoxanide (TIZ), the circulating metabolite of NTZ, also inhibited MeV infection, further confirming the results (Suppl. Fig. 1).

**Table 1:** IC_50_ and selectivity index of nitazoxanide in measles virus (MeV) and canine distemper virus (CDV) infections..

Virus	IC_50_	CC_50_	Selectivity index
MeV	3.23 μg/ml	>50 μg/ml	>15.5
CDV	2.83 μg/ml	>50 μg/ml	>17.7

Altogether, these results show that NTZ is very effective against *Morbillivirus* infection at a post-entry level.

**Suppl. Fig.2:**
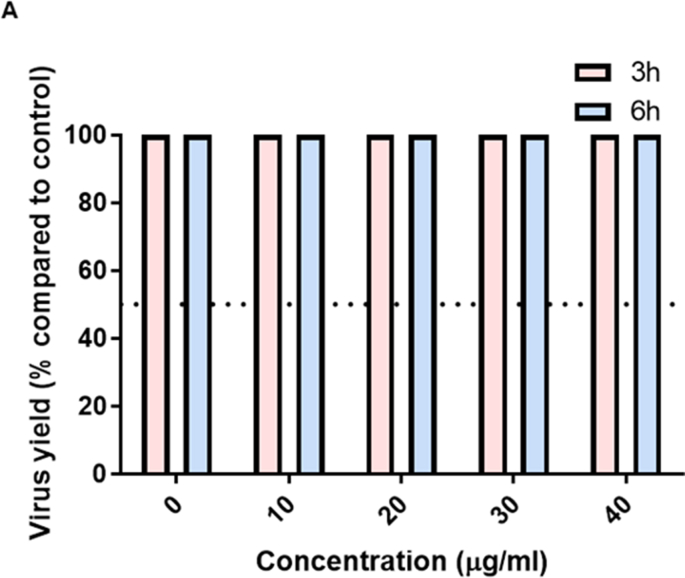
Pretreatment of cells with NTZ does not affect MeV infection. Cells were treated with the indicated concentrations of NTZ for 3 or 6 h before infection with measles virus. After drug removal cells were washed three times with PBS, and infected with MeV for 2 h, after which time carboxymethylcellulose was added. At 72 h p.i., cells were fixed with 4% paraformaldehyde and the plaques were stained using crystal violet. Results of a representative experiment are shown.

In line with previously reported data on the Sendai virus, NTZ did not show significant antiviral activity when cells were pre-treated for 3 or 6 h before infection (Suppl. Fig. 2).

### 2.2 NTZ inhibits viral spread

**Fig. 2:**
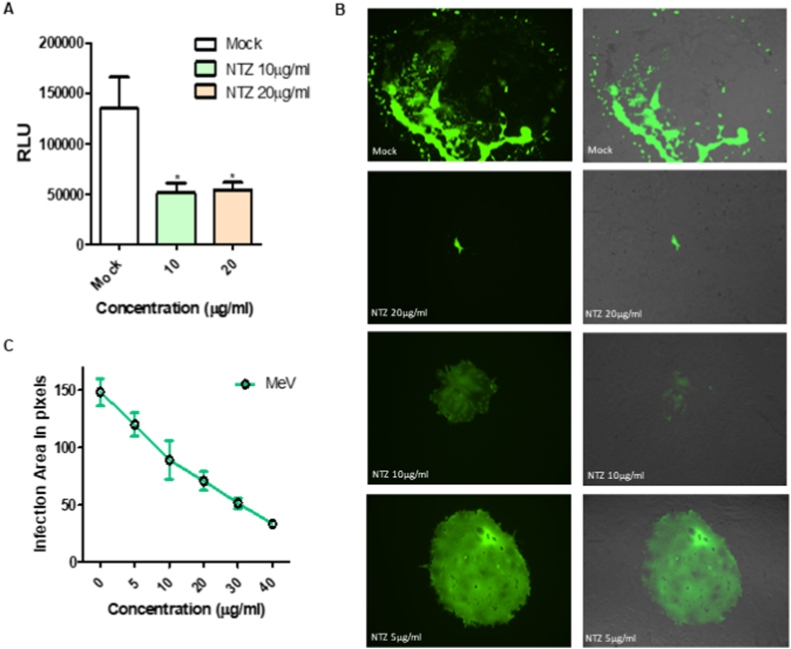
Effect of nitazoxanide (NTZ) on viral fusion and spread. (A) The fusion assay was performed in the presence or in the absence of NTZ (10 or 20 μg/ml) and in HEK-293T cells co-transfected with the measle virus (MeV) IC323–F-wt and MeV IC323–H-wt. Target cells were transfected with nectin-4 as a receptor. Effector cells were overlaid on target cells expressing the nectin-4 receptor and incubated overnight. NTZ was added to target cells 30 min before adding the effector cells. (B)PVero-hSLAM cells were infected with MeV IC323-EGFP in the presence of the indicated concentrations of NTZ. 72 h p.i the cells were fixed with 4 % paraformaldehyde, images were collected with a Nikon Ti2–U fluorescent microscope and the area of infection was measured in pixels using ImageJ software. (C) Graph showing the infection area in pixels measured with ImageJ software. * p-value<0.05.

It has been previously reported that, in the case of the paramyxovirus SeV, NTZ acts as a non-competitive inhibitor of ERp57, a thiol oxidoreductase that is required for the proper folding of the viral fusion protein F.^22^ NTZ treatment was found to alter the F glycoprotein architecture, leading to the formation of F aggregates, in turn impairing its function.^22^ We have speculated that, similarly to SeV, an altered F function in NTZ-treated cells could impact the ability of the *Morbillivirus* F glycoprotein to promote the fusion process required for the entry and spread of MeV. We have therefore evaluated the ability of the measles F protein to promote fusion after NTZ treatment. As shown in Fig. 2A, in the presence of NTZ the extent of F-mediated fusion is significantly decreased.

**Suppl. Fig.3:**
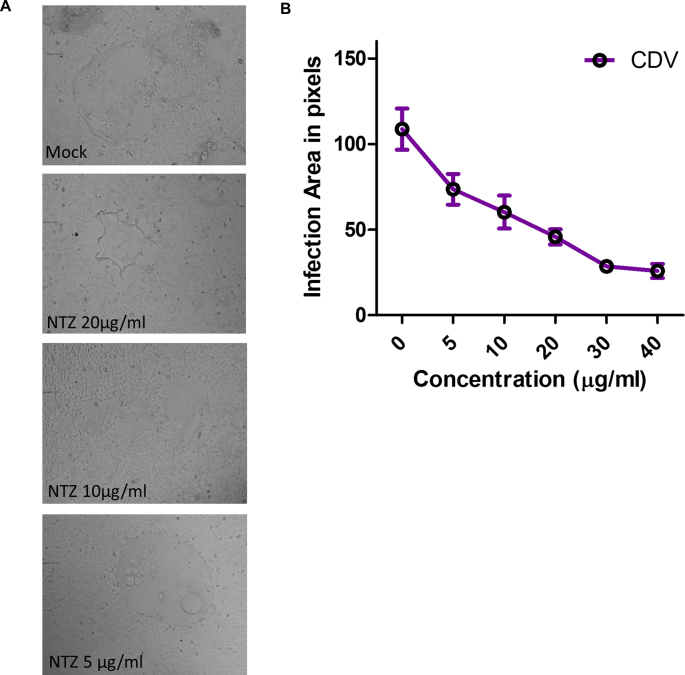
Decrease of CDV spread in the presence of NTZ. (A) Vero-hSLAM cells infected with CDV in the presence of different concentrations of NTZ. Images were randomly acquired with a Nikon Ti2-U inverted microscope and the area of infection in pixels was measured using ImageJ software. (B) Graph showing the infection area in pixels measured with ImageJ software.

MeV and CDV are mainly cell-associated viruses. Since the F protein plays a key role in cell-to-cell spread, we have assessed whether NTZ impacted viral spread. As shown in Fig. 2B–C, NTZ treatment strongly reduced the size of viral plaques during MeV infection. Even at 5 μg/ml NTZ prevented the extensive monolayer tearing observed in the untreated control. Similarly, we have observed a decreased area of infection when CDV replication occurred in the presence of NTZ (Suppl. Figs. 3A and 3B). Together these results suggest that NTZ blocks infection by limiting viral spread.

## 3 Discussion

Despite a global effort to eradicate measles infection, MeV is far from being eliminated, and we face a resurgence of measles in several countries.^28^, ^29^, ^30^ In 2021 MeV caused more than 120.000 deaths.^31^ In the same year, the percentage of children who has received two doses of vaccine worldwide had dropped to 71%.^31^ This percentage is far below the 95% required to obtain herd immunity and prevent the transmission of measles virus in the community. Vaccine hesitancy due to parents’ safety concerns about this live-attenuated vaccine, combined with the COVID-19 pandemic. have fuelled measles spread globally. Indeed, the suspension of immunization services during the early phase of the SARS-CoV-2 pandemic caused a decrease in immunization rates.^31^ This, combined with reduced worldwide surveillance, has made millions of children susceptible to measles infections.

A global effort is required to boost measles vaccination rates and implement surveillance systems. A therapeutic strategy to complement the vaccination campaign would complement measles eradication efforts and help contain outbreaks. Additionally, a drug could fill an unmet clinical need for existing measles complications, which can be extremely serious and include pneumonia and lethal brain diseases.^32^ Neurological sequelae of MeV infection include measles inclusion body encephalitis (MIBE) and subacute sclerosing panencephalitis (SSPE). MIBE occurs mainly in immunocompromised individuals within 1 year of acute measles infection.^33^ SSPE may appear several years after primary infection in immunocompetent subjects. Unfortunately, no specific therapy is currently available for MIBE and SSPE diseases.

Like MeV, CDV may also cause neurological complications, but with higher frequency. CNS involvement is common in infected dogs, but the clinical manifestations strongly depend on the immune status of the animal. Dogs with low antibody response frequently show acute or chronic demyelination. For these reasons the development of an effective therapeutic approach against morbilliviruses is an important goal.

As previously mentioned, NTZ is a broad-spectrum FDA-approved antiviral drug that is effective in the treatment of rotavirus gastroenteritis, airway infections from influenza, and other respiratory viruses including Paramyxoviruses and Coronaviruses.^19^, ^20^, ^21^, ^22^^,^^27^

Clinical trials have shown that in children and adults with rotavirus infection, NTZ treatment reduced the duration of symptoms of severe diarrhea.^24^ In the case of influenza, a phase 2b/3 trial showed that NTZ reduced the duration of symptoms in patients with acute uncomplicated influenza.^26^ Recently, clinical trials of nitazoxanide alone or in combination with antivirals against SARS-CoV-2 have shown antiviral activity and clinical benefits of NTZ treatment in COVID-19 patients.^25^^,^^34^, ^35^, ^36^, ^37^, ^38^

Piacentini and colleagues^22^ previously demonstrated the potent antiviral activity of nitazoxanide against the Paramyxovirus Sendai.

In this study, we have investigated the potential antiviral activity of nitazoxanide and its metabolite tizoxanide on *Morbillivirus* infection. We found that NTZ exerts a strong antiviral activity against MeV and CDV at low μM concentrations that can be achieved in the plasma of NTZ-treated patients.^26^ NTZ potently inhibits viral replication when treatment is started after virus adsorption, indicating that NTZ acts at post-entry level, as previously observed for other members of the *Paramyxoviridae* family. A weak antiviral activity was observed in short-term (2 h) NTZ treatment during viral adsorption and only at very high concentration, while, as previously shown in the case of Sendai virus,^22^ viral replication was not significantly affected in cells pre-treated with NTZ for 3 or 6 h before infection.

In *Paramyxoviridae* the F protein is essential for virus entry and cell-to-cell spread. Indeed, the F glycoprotein promotes the direct fusion of viral and cell membranes, allowing the virus to enter the cells and facilitating viral spread between cells. As previously mentioned, nitazoxanide was shown to selectively impair the maturation and intracellular transport of the fusion protein of the Paramyxovirus SeV and of RSV, an effect associated with the drug-mediated inhibition of ERp57, an ER-resident thiol oxidoreductase required for the correct disulfide-bond architecture of selected viral proteins.^22^ We therefore investigated whether NTZ treatment also affected *Morbillivirus* F protein activity. We observed that, in fact, the extent of MeV–F-mediated cell-cell fusion decreased after NTZ treatment, suggesting an effect of the drug on the F protein in this case. *Morbilliviruses* are mainly cell-associated viruses that spread from cell-to-cell by leveraging F-mediated fusion. Cell-to-cell spread has several advantages, including the evasion of antibody-mediated immune response and the by-passing of the epithelial barrier.^39^ Cell-to-cell spread is also a key feature for the dissemination and persistence of measles in the CNS.^40^^,^^41^

The fact that NTZ treatment reduced both the number of plaques and the area of infection suggests that the drug may impair F-mediated cell-to-cell spread.

## 4 Conclusion

Currently no approved antiviral agents are available against MeV and CVD. We show here that nitazoxanide, approved for clinical use as a safe and effective antiprotozoal drug, inhibits both MeV and CDV replication *in vitro*, impairing the function of the fusion protein and blocking viral spread. Altogether, the results suggest that nitazoxanide should be explored as a potential antiviral for acute and persistent MeV and CVD infections.

## 5 Materials and methods

### 5.1 Cell culture and treatments

HEK-293T (Human kidney epithelial) and Vero-human-SLAM (Vero-hSLAM, African green monkey kidney) cells obtained from American Type Culture Cell Collection (ATCC), were grown at 37 °C in 5% CO_2_ atmosphere in Dulbecco's modified Eagle's medium (DMEM; Gibco, cat. num. 11995065) supplemented with 10% fetal bovine serum (FBS; Gibco, cat. num. 10270106) and antibiotics (Gibco, cat. num. 15140122). Vero-hSLAM media was supplemented with geneticin 0.4 mg/ml (Gibco, cat. num. 10131035). Nitazoxanide [(2-acetyloxy-N-(5-nitro-2-thiazolyl)benzamide] and tizoxanide (N0290-10 MG, Sigma-Aldrich; T450100 1 Tizoxanide, Toronto Research Chemicals), dissolved in dimethyl sulfoxide (DMSO, 10 mg/ml) stock solution, were diluted in cell culture medium, added to the infected and mock-infected cell, and maintained in the medium for the entire duration of the experiment. Controls received equal amounts of DMSO, which did not affect cell viability or virus replication.

### 5.2 Cell viability

Cytotoxicity of the thiazolides was determined by assessing the conversion of MTT [3-(4,5-dimethylthiazol-2-yl)-2,5-diphenyltetrazolium bromide] (MTT) to MTT-formazan as described in the manufacturer's protocol (Vybrant® MTT Cell Proliferation Assay Kit, Cat. No. V-13154). Briefly, Vero-hSLAM cells were treated with NTZ or TIZ at concentrations ranging from 5 to 200 μg/ml for 72 h. After that, MTT (10 μl of 12 mM stock solution in 100 μl of medium/well) was added to the cells and incubated at 37 °C in a humidified atmosphere. After 4 h, 50 μl of DMSO were added in each well and the plate incubated at 37°C for 10 min. MTT conversion in formazan was assessed measuring the absorbance at 540 nm using TECAN instrument. The 50% cytotoxic concentration (CC_50_) and inhibitory concentration (IC_50_) were calculated using GraphPad Prism software to determine the Selectivity Indexes of the thiazolides.

### 5.3 Plasmids and transfections

Sequences encoding measles fusion and attachment proteins (MeV F and H, GenBank: LC420351.1) and SLAM (GenBank: U33017.1) and Nectin-4 cellular (GenBank: AB755430.1) receptors were codon optimized, synthesized, and subcloned into the mammalian expression vector pCAGGS by Epoch Biolabs. Transfections were performed using Lipofectamine™ 2000 Transfection Reagent (Invitrogen, cat. num. 11668019), according to the manufacturer's protocol.

### 5.4 Cell-to-cell fusion assay

Cell-to-cell fusion was evaluated through a fusion assay based on the complementation of β-galactosidase (β-Gal) as previously described.^42^^,^^43^

Briefly, HEK-293T cells co-expressing nectin-4 receptor and the omega subunit of β-galactosidase were incubated with cells transiently transfected with plasmids encoding measles F, H and the alpha reporter subunit. The activity of the reconstituted β-galactosidase is proportional to the extent of fusion. Cells were lysed and the enzymatic activity was quantified using the Galacton-Star kit (Applied Biosystems) and the Infinite M1000PRO (Tecan) microplate reader.

### 5.5 Virus, infection and titering

MeV IC323-EGFP is a recombinant virus expressing the EGFP gene, located between the leader and the N sequence.^44^ The recombinant virus was generated using a plasmid encoding for IC323 MeV sequence generously provided by Yusuke Yanagi, Kyushu University, Japan.^44^ Canine distemper virus (Ondersteport strain) was obtained by ATCC.

For co-treatment assay 1.5x10^5^ of Vero-hSLAM cells were seeded in 12-well plate. After 24 h, the indicated concentrations of nitazoxanide or tizoxanide were mixed with the virus (100 PFU) and the mix was added on top of the cells. Two hours later, nitazoxanide was removed, the cells were washed three times with PBS and then media containing 3 % carboxymethylcellulose was added. The cells were incubated at 37 °C for 72 h. After that, the plates were fixed with 4 % paraformaldehyde and stained with crystal violet.

For the post-infection assay, Vero-hSLAM monolayers were infected with 100 PFU/well of MEV o CDV for 2 h (multi-step growth). After the adsorption step, the virus was removed, and cells maintained in a medium containing 5 % FBS in the presence or absence of thiazolide treatment. After 72 h, the supernatants were collected following multiple freeze/thaw cycles and virus yield was determined by plaque assay.

### 5.6 Cell pre-treatment

Vero-hSLAM cells were seeded in 12-well plate the day before the infection. After 24 h, cells were treated with different concentrations of nitazoxanide for 3 and 6 h. After the pre-treatment period the cells were washed three times with PBS and then infected with 100 PFU of CDV or Measles for 2 h to allow viral adsorption; the virus was then removed and plaque assays were performed as previously described.

### 5.7 Plaques enlargement assay

To assess the cell-to-cell viral spread we measured the infection area in pixels as previously described.^45^ Briefly, Vero-hSLAM cells were plated in 12 well-plate (1.5x10^5^ cells/well). The following day, the cells were infected with 100 PFU of MeV IC323-EGFP or CDV for 2 h at 37 °C. The media was replaced with media containing 3 % carboxymethylcellulose and NTZ at different concentrations. After 72 h, cells were fixed with 4 % paraformaldehyde and the infected cells were imaged using Nikon Ti2–U inverted microscope (20x objective). The area of infection was quantified using ImageJ software.

### 5.8 Statistical analysis

Statistical analysis was performed using GraphPad Prism 8 (GraphPad Software). Data represent the means ± standard errors of the means (SEM) from at least three independent experiments. For statistical analysis Student's one-tailed *t*-test was applied. *P value < 0.05, **P value < 0.01, ***P value < 0.001.

## Funding

The authors are grateful to PRIN 2017 “Natural and pharmacological inhibition of the early phase of viral replication (VirSudNet)” No 2017M8R7N9 and the Valere project of the University of Campania “Luigi Vanvitelli” for the support in publishing this article. NIH support to MP (AI159085, NS091263, and NS105699).

## Authors' contributions

DS and MP designed the experiments. DS and AA carried out the experiments. DS, SLF and MG wrote the manuscript. MP, MGS, CZ, ADF, DT, VI and SM revised the manuscript. All authors contributed to the interpretation of the data and approve the content of the manuscript.

## Declaration of competing interest

The authors declare that they have no known competing financial interests or personal relationships that could have appeared to influence the work reported in this paper.

## Data Availability

Data will be made available on request.

## List of abbreviations

NTZ: (nitazoxanide)
TIZ: (tizoxanide)
MeV: (measles)
CDV: (canine distemper virus)
PPRV: (peste des petits ruminants virus)

## Footnotes

Appendix A Supplementary data to this article can be found online at https://doi.org/10.1016/j.jve.2023.100353.
